# Characterizing defective lipid metabolism in the lateral septum of mice treated with olanzapine: implications for its side effects

**DOI:** 10.3389/fphar.2024.1419098

**Published:** 2024-06-14

**Authors:** Lixuan Huang, Ying Sun, Chao Luo, Wei Wang, Si Shi, Genmin Sun, Peijun Ju, Jianhua Chen

**Affiliations:** ^1^ Shanghai Mental Health Center, Shanghai Jiao Tong University School of Medicine, Shanghai, China; ^2^ Shanghai Key Laboratory of Psychotic Disorders, Shanghai Mental Health Center, Shanghai, China; ^3^ Shanghai Institute of Traditional Chinese Medicine for Mental Health, Shanghai, China; ^4^ Yueyang Hospital of Integrated Traditional Chinese and Western Medicine, Shanghai University of Traditional Chinese Medicine, Shanghai, China; ^5^ First Affiliated Hospital of Xinjiang Medical University, Urumqi, China

**Keywords:** olanzapine, lateral septum, functional ultrasound imaging, RNA sequencing, WGCNA

## Abstract

Schizophrenia significantly impacts cognitive and behavioral functions and is primarily treated with second-generation antipsychotics (SGAs) such as olanzapine. Despite their efficacy, these drugs are linked to serious metabolic side effects which can diminish patient compliance, worsen psychiatric symptoms and increase cardiovascular disease risk. This study explores the hypothesis that SGAs affect the molecular determinants of synaptic plasticity and brain activity, particularly focusing on the lateral septum (LS) and its interactions within hypothalamic circuits that regulate feeding and energy expenditure. Utilizing functional ultrasound imaging, RNA sequencing, and weighted gene co-expression network analysis, we identified significant alterations in the functional connection between the hypothalamus and LS, along with changes in gene expression in the LS of mice following prolonged olanzapine exposure. Our analysis revealed a module closely linked to increases in body weight and adiposity, featuring genes primarily involved in lipid metabolism pathways, notably *Apoa1*, *Apoc3*, and *Apoh*. These findings suggest that olanzapine may influence body weight and adiposity through its impact on lipid metabolism-related genes in the LS. Therefore, the neural circuits connecting the LS and LH, along with the accompanying alterations in lipid metabolism, are likely crucial factors contributing to the weight gain and metabolic side effects associated with olanzapine treatment.

## 1 Introduction

Schizophrenia is a debilitating mental disorder that emerges in early adulthood and significantly impairs cognitive and behavioral functions (Solmi et al., 2023). According to current clinical guidelines, the treatment regimen primarily includes second-generation antipsychotics (SGAs) such as olanzapine, clozapine, and risperidone. Although SGAs represent a cornerstone in schizophrenia management, their usage is marred by significant metabolic side effects. These include weight gain, dyslipidemia, and insulin resistance. These effects further diminish patient compliance, exacerbate psychiatric symptoms, and heighten the risk of cardiovascular diseases and mortality (Correll et al., 2014; Vancampfort et al., 2015; Hirsch et al., 2017; Vantaggiato et al., 2019). Peripheral metabolic abnormalities have recently been recognized to be regulated by the central nervous system (Myers and Olson, 2012; Ruud et al., 2017; Seicol et al., 2019; Shen et al., 2024). The possibility that these atypical antipsychotic drugs may influence the molecular determinants of synaptic plasticity and brain activity while exerting their therapeutic effects also affects the central response to the side effects of these drugs (Ibi et al., 2017; de Bartolomeis et al., 2022). Current understanding of the adverse effects induced by olanzapine indicates a complex interplay among brain activity, particularly involving the hypothalamic area and its interaction with other brain regions, though these mechanisms necessitate further clarification (Martins et al., 2010; Stanley et al., 2010; Chen et al., 2022; Huang et al., 2022; Fang et al., 2023; Meng et al., 2023). It is highly worthwhile to explore the changes in central network functional connectivity corresponding to the induction of peripheral obesity and metabolic side effects by olanzapine.

Currently, it is known from a sample of patients with schizophrenia that olanzapine can modulate long- and short-range functional connectivity in the resting brain (Guo et al., 2017). The regulation of metabolism is intricate, extending beyond isolated brain regions to encompass complex neural circuits. The hypothalamic neurocircuits have garnered lots of attention. The hypothalamic neurocircuits, extensively researched and crucial for central regulation of feeding and energy expenditure, are intricately linked to the mesolimbic reward system, governing the hedonic aspects of food consumption and weight gain (Timper and Brüning, 2017). Brain areas such as the lateral hypothalamus (LH), lateral septum (LS), ventral tegmental area (VTA), and nucleus accumbens (NAcc) have been implicated in the homeostatic and hedonic control of feeding in experimental models of diet-induced obesity (Marcos et al., 2023). Among them, the LSv functions as a most important node in mediating melanocortin action on body-weight regulation. It is known that such as the LS region, suggests a more comprehensive system of metabolic control (Terrill et al., 2016; Terrill et al., 2018; Calderwood et al., 2022; Gabriella et al., 2022). The LS influences feeding behavior, aggression, food seeking, and stress responses, with emerging evidence pointing to its involvement in weight regulation through hypothalamus-LS circuit and its potential susceptibility to SGA effects (Wong et al., 2016; Carus-Cadavieco et al., 2017; Xu et al., 2023b; Wang et al., 2023). Therefore, exploring the involvement of LS in central side effects of Olanzapine is of significant importance.

Our study aims to elucidate the intricate and long-term metabolic effects of olanzapine, with a specific focus on the LS region in mediating these effects. We propose that olanzapine exerts its influence on metabolic function and weight regulation by modulating the LS area, thereby contributing to the adverse metabolic outcomes observed in schizophrenia treatment. Employing advanced methodologies such as functional ultrasound imaging (fUS) to investigate LS-hypothalamus connectivity, and utilizing RNA sequencing combined with weighted gene co-expression network analysis (WGCNA) (Zhang and Wong, 2022), our investigation explores the genetic landscape altered by olanzapine in the LS region. Our results demonstrate that prolonged exposure to olanzapine disrupts LS-hypothalamus connectivity and alters gene expression associated with lipid metabolism in the LS region, highlighting the involvement of key genes such as *Apoa1*, *Apoc3*, and *Apoh* in these processes. By elucidating the central role of olanzapine in modulating LS-hypothalamus dynamics and gene expression patterns related to lipid metabolism, our findings not only enhance understanding of SGA-induced metabolic disturbances but also lay the groundwork for potential therapeutic interventions aimed at mitigating these side effects.

## 2 Materials and methods

### 2.1 Study design

This research aimed to elucidate the role of olanzapine in inducing weight gain and related metabolic dysfunctions, with a particular focus on its impact on the LS region of the brain. Using a mouse model to replicate the side effects of antipsychotic drugs observed in humans, we specifically focused on female mice due to their distinct response to olanzapine, as highlighted in previous research. Retrospective clinical studies have identified the female gender as a predictor for antipsychotic-induced weight gain with atypical antipsychotics (Gebhardt et al., 2009). Animal studies have also shown that female rats experience more significant weight changes after long-term olanzapine use compared to male rats (Choi et al., 2007). Age-matched female mice were randomized into two groups (n = 15 per group) to receive either an olanzapine-supplemented diet or a standard chow diet. Body weight was monitored weekly as a measure of olanzapine’s effects, with the aim of minimizing animal stress. The study duration was 12 weeks, with a significant weight gain checkpoint at 8 weeks. At the end of the treatment, we randomly selected nine mice from each group for body composition measurement, serum lipoprotein analysis and scRNA-seq, while the remaining six underwent fUS scanning. LS brain regions in this cohort of mice were used to validate the results of scRNA-seq.

### 2.2 Animals

All animal experimental protocols were approved by the Institutional Animal Care and Use Committee of Shanghai Mental Health Center. Mice were housed in an SPF-grade experimental animal room under standard laboratory conditions (12-h on/off; lights on at 7:00 a.m.) in a temperature-controlled environment with food and water available *ad libitum*. Female C57BL/6 mice were obtained from Gempharmatech Co., Ltd (Nanjing, China) at about 8 weeks of age.

### 2.3 Animal treatment

After 1 week of acclimation in the animal room, female mice were placed either on control chow diet (maintenance feed) or olanzapine (PHR 1825, Merck, Germany) diet (50 mg/kg in maintenance feed). Olanzapine dosage referenced from previous literature maintains effective blood level (Morgan et al., 2014). Feed for the mice was customized and purchased from Jiangsu Xietong Pharmaceutical Bio-engineering Co., Ltd. (Nanjing, China). Body weight was monitored on each Friday morning. Feeding was continued for 12 weeks.

### 2.4 Preparation for fUS

fUS preparation is implemented with reference to previous literature (Brunner et al., 2021). Mice designated for fUS testing underwent a preparatory procedure that began with anesthesia, using isoflurane for both induction (3%) and maintenance (1%) to ensure minimal discomfort. Following anesthesia, the scalp was carefully removed, and tissues were cleared to fully expose the cranial surface. A custom stainless steel head locator was then securely attached to the skull using dental adhesive to facilitate precise positioning during fUS testing. The skull’s surface was meticulously thinned using a specialized cranial drill and grinder. Care was taken to avoid any abrasion to the skull, ensuring the integrity of the cranial structure was maintained. After the cranial preparation, an ultrasound coupling agent was applied to keep the skull moist, followed by a protective covering of dental silicone light body. Mice were given a 3-day recovery period post-surgery, during which they received careful monitoring and pain management as needed. To acclimatize the mice to the fUS assay setup, they were placed in a fixation frame for 1 h daily. This step was critical to reduce stress and ensure accurate and consistent fUS measurements.

### 2.5 fUS procedures

We utilized a fUS scanning protocol based on established methodologies as detailed in prior studies (Grohs-Metz et al., 2022). The imaging was conducted using specialized small animal fUS equipment provided by Iconeus (Paris, France), which included a linear ultrasonic probe (15 MHz central frequency) with 128 piezoelectric transducers. This probe was integrated with the ultrafast Iconeus One ultrasound scanner (128 channels), facilitating the emission and reception of ultrasonic plane waves, as well as the processing of the resulting Power Doppler signals. The clutter filtering technique applied for the isolation of blood signals was performed via spatiotemporal singular value decomposition, a method thoroughly documented in the literature. The compiled fUS images were derived from 200 compounded ultrafast frames, acquired at a rate of 500 Hz. Each frame aggregated the ultrasonic echoes from a series of 11 tilted plane waves (ranging from −10 to 10°, with 2-degree intervals) emitted at a pulse repetition frequency of 5,500 Hz. The final image sequence was produced at a frame rate of 2.5 Hz, translating to one Power Doppler image every 400 milliseconds, and featured a spatial resolution of 100 × 100 × 400 μm. For the scanning procedure, once the animals were secured and their scalps prepared and treated with isotonic coupling gel, the ultrasonic probe was positioned approximately 1 mm above the scalp to ensure full immersion in the gel. The probe’s location was determined using Iconeus Studio software to target an oblique plane, which encompassed the neuroanatomical regions of interest (ROIs) associated with the LS and hypothalamus. 20 min of Power Doppler images were captured for each session. The initial 15 min served as an acclimatization period to calm the mice, followed by a 5-min official recording phase utilized for the analysis.

### 2.6 Tissue harvesting and body composition measurement

Mice were terminally anesthetized with isoflurane and 1% chloralhydrate. After anesthesia, 200ul blood samples were collected from the mice by orbital blood sampling. LS tissues were micro-dissected under a surgical microscope and then flash-frozen in liquid nitrogen. The rest of body were stored on ice and send to get body composition measurement. Fat and lean mass of mice body were detected using an EchoMRI-100H Body Composition Analyzer (EchoMRI, USA) with EchoMRI 2016 software.

### 2.7 Serum processing and lipid profiling

The serum was separated by centrifugation at 900 *g* for 5 min at 4°C using a Centrifuge 5430 R (Eppendorf, Germany), 1 hour after blood collection. Total cholesterol (TC), triglycerides (TG), high-density lipoprotein cholesterol (HDL-C), and low-density lipoprotein cholesterol (LDL-C) levels were quantified using enzyme-linked immunosorbent assay (ELISA) kits (Nanjing Jiancheng Bioengineering Institute), following the manufacturer’s instructions.

### 2.8 RNA isolation and library preparation

Due to the small size of the LS region, samples from each three mice were pooled to ensure sufficient RNA volume for sequencing, addressing the challenge of low tissue yield. Total RNA extraction from pooled LS regions used TRIzol reagent (Invitrogen, CA, USA), with purity and concentration verified by NanoDrop 2000 (Thermo Scientific, USA). RNA integrity was assessed with an Agilent 2,100 Bioanalyzer. The VAHTS universal V6 RNA-seq Library Prep Kit was utilized for library construction, with OE Biotech Co., Ltd. (Shanghai, China) performing transcriptome sequencing and analysis.

### 2.9 RNA-seq and differentially expressed genes analysis

The sequencing of libraries was carried out on an Illumina NovaSeq 6,000 platform, producing 150 bp paired-end reads. Each sample yielded approximately 51.74 million raw reads. These raw reads, in FASTQ format, were initially processed with fastp (Chen et al., 2018) to remove low-quality reads, resulting in clean reads. Subsequently, around 48.78 million clean reads per sample were obtained for further analysis. Mapping of clean reads to the reference genome was conducted using HISAT2(Kim et al., 2015), followed by the calculation of the fragments per kilobase of transcript per million mapped reads (FPKM) (Roberts et al., 2011) for each gene. Gene read counts were determined using HTSeq-count (Anders et al., 2015). To assess the replicability of biological samples, PCA (Principal Component Analysis) was carried out using R (version 3.2.0). Differential gene expression analysis was executed using DESeq2 (Love et al., 2014), with a Q value of <0.05 and a fold change >2 or <0.5 as criteria for significant differential expression. Hierarchical clustering analysis of differentially expressed genes (DEGs) was conducted in R to illustrate the gene expression patterns across various groups and samples. Enrichment analyses for Gene Ontology (GO) and Kyoto Encyclopedia of Genes and Genomes (KEGG) pathways of the DEGs were performed based on the hypergeometric distribution, using R to identify significantly enriched terms. R was also employed to generate column diagrams, chord diagrams, and bubble diagrams to visually represent the significant enrichment terms.

### 2.10 WGCNA of RNA-seq data

WGCNA was conducted utilizing the FPKM expression dataset with the WGCNA package in R (Langfelder and Horvath, 2008). Genes exhibiting variance values exceeding 25% were excluded, resulting in a selection of genes with high expression levels for further analysis. Module identification was achieved through the application of a tree-cutting algorithm. Traits such as weight gain, TC, TG, HDL-C, LDL-C, fat mass, and lean mass were incorporated as diagnostic markers for assessing weight and metabolic changes. These traits facilitated the correlation analysis with identified gene modules. Functional annotation of the modules was performed using the org. Hs.e.g.,.db annotation package. Enrichment analyses for GO and KEGG pathways were executed employing the clusterProfiler package in R (Yu et al., 2012), providing insights into the biological functions and pathways associated with the genes within the significant modules.

### 2.11 Identify the known targets of olanzapine

To identify the known gene targets of olanzapine, we utilized the GeneCards database^1^. GeneCards serves as an exhaustive, searchable repository that amalgamates information from approximately 150 gene-centric databases. It encompasses a wide array of functional data essential for the annotation and prediction of human genes, alongside comprehensive genomics, transcriptomics, and proteomics data. The search for relevant gene targets was conducted by entering “olanzapine” as the keyword, facilitating a targeted exploration of the database’s extensive resources.

### 2.12 Protein-protein interaction (PPI) network construction and analysis

For the purpose of delineating the PPI network encompassing both the modules and the overlapping targets between these modules and the established targets of olanzapine, we engaged the STRING database^2^. This database is renowned for its comprehensive compilation of PPI data, derived from both empirical research and computational predictions. Within this framework, the confidence level of each interaction is quantitatively expressed through a score, which spans from 0 to 1, facilitating a nuanced assessment of interaction reliability. Subsequently, genes common to both the modules of interest and the known targets of olanzapine were imported into the analysis. The raw PPI network data thus obtained was then transferred to Cytoscape 3.10.1 for further examination. Utilizing the topological parameter known as “degree,” we conducted an analysis to discern the central targets within the network. These targets, ranked according to their degree values, were then extracted to construct a focused visualization of the PPI network. This visualization highlighted the central targets, thereby offering a refined view of the main PPI network and elucidating potential key players in the biological processes influenced by olanzapine.

### 2.13 Quantitative reverse transcription polymerase chain reaction (qRT-PCR)

The LS tissues were processed using a rotor–stator homogenizer for disruption and homogenization. Total RNA was extracted from the frozen, dissected LS tissues employing the phenol-chloroform extraction method. We converted 400 ng of total RNA into complementary DNA (cDNA) utilizing the RevertAid First Strand cDNA Synthesis Kit (91255466, Thermo Fisher Scientific, USA). The relative mRNA expression levels of Actin, Apoa1, Apoc3, and Apoh were quantified through qRT-PCR, employing TB Green Premix Ex Taq II (AMG1484A, Takara Bio, Japan). The fluorescence of TB Green was detected and analyzed using the LightCycler480 system (Roche Diagnostics, Switzerland). Gene expression was normalized against the expression of mouse Actin mRNA. The 2^(−ΔΔCt) method was utilized for the relative quantification of gene expression, with results presented as log (2^(−ΔΔCt)). Primer sequences used in the analysis were as follows: *Actin* forward: GCT​CTT​TTC​CAG​CCT​TCC​TT, *Actin* reverse: TGA​TCC​ACA​TCT​GCT​GGA​AG, *Apoa1* forward: GGC​AGA​GAC​TAT​GTG​TCC​CAG​TT, *Apoa1* reverse: CCC​AGT​TTT​CCA​GGA​GAT​TCA​G, *Apoc3* forward: CAT​CTG​CCC​GAG​CTG​AAG​A, *Apoc3* reverse: GCT​TGT​TCC​ATG​TAG​CCC​TGT​AC, *Apoh* forward: GCA​GAG​ATG​GCA​CTA​TCG​AGA​TTC​C, *Apoh* reverse: CGA​CTT​CAG​CAC​GGT​GTC​AGT​TC.

### 2.14 fUS data processing

Temporal Power Doppler signals were extracted from ROIs defined using the Allen Mouse Brain Common Coordinate Framework (Wang et al., 2020), with alignment to individual animals and imaging sessions, as detailed in prior studies (Nouhoum et al., 2021). Signals from all voxels within an ROI were normalized and then detrended using a fourth-order polynomial to mitigate low-frequency drifts. Subsequently, a low pass filter with a cutoff at 0.1 Hz was applied to retain only the frequencies relevant to resting-state functional connectivity. Connectivity matrices were constructed by calculating the Pearson product-moment correlation coefficients between ROI pairs. For comparative analysis of functional connectivity among different brain regions, Pearson’s r-values underwent Fisher’s z transformation for normalization, followed by subsequent statistical evaluation.

### 2.15 Statistical analysis

Data analysis and visualization were performed using GraphPad Prism eight software (GraphPad Software, USA). Results are presented as mean ± standard deviation (SD). Two-way ANOVA followed by Sidak’s multiple comparisons test was utilized for repeated measures such as weight gain. The Geisser-Greenhouse correction was applied to ensure variance homogeneity. The Shapiro-Wilk test was employed to verify data distribution normality, and variance homogeneity was assessed via the F-test. Variables that followed a normal distribution and exhibited homogeneity of variances were analyzed using the independent samples t-test, while the Mann-Whitney U test was used for variables that did not conform to normal distribution. When variance homogeneity was not achieved, Welch’s correction was applied. Statistical significance was determined by a two-tailed test with a significance threshold set at *p* < 0.05.

## 3 Results

### 3.1 Screening for specific brain functional connectivity associated with obesity in mice treated with olanzapine

**FIGURE 1:**
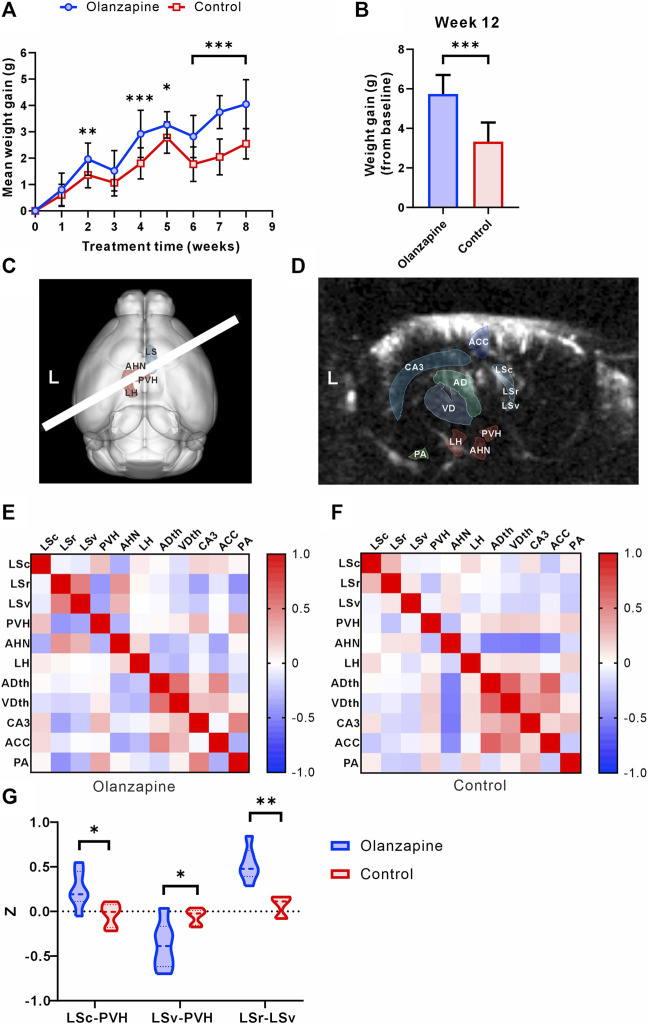
Results of statistical analysis for weight gain and functional connectivity changes between two groups. **(A)** Body weight gain of mice from olanzapine group (Olanzapine) and control group (Control) during first 8 weeks of treatment. N = 15 per group. Sidak’s multiple comparisons test after Geisser-Greenhouse correction. **(B)** Body weight gain measured at the end of 12 weeks treatment. N = 15 per group. Unpaired t-test. F = 1.004, *p* < 0.001, R^2^ = 0.6266, t = 6.854, degrees of freedom (df) = 28. **(C)** 3D registration enabled linear probe positioning (white bar) through the LS (red region) and hypothalamus (cyan region). **(D)** Anatomical delineations derived from the Allen Common Coordinate Framework overlaid on a representative fUS Doppler image show coverage of ROIs. **(E)** Heatmap of Pearson correlation coefficient between selected ROIs in olanzapine group. **(F)** Heatmap of Pearson correlation coefficient between selected ROIs in control group. **(G)** Differences in functional connectivity z-values between olanzapine and control groups for LSc-PVH (Unpaired t-test, F = 2.637, *p* = 0.0192, R^2^ = 0.4370, t = 2.786, df = 10), LSv-PVH (Welch’s t-test, F = 9.488, *p* = 0.0309, R^2^ = 0.5468, t = 2.800, df = 6.042) and LSc-LSv (Mann-Whitney U test, *p* = 0.0022). N = 6 per group. The data are shown as mean ± SD. *p < 0.05, **p < 0.01, ***p < 0.001. LSc lateral septum caudal part, LSr lateral septum rostral part, LSv lateral septum ventral part, LH lateral hypothalamic area, AHN anterior hypothalamic nucleus, PVH paraventricular hypothalamic nucleus, ACC anterior cingulate cortex, CA3 hippocampal CA3 region, ADth anterior group of the dorsal thalamus, VDth ventral group of the dorsal thalamus, PA posterior amygdala nucleus.

At the outset, the study groups were comparable in terms of body weight. However, over the course of the 8-week observation period, mice treated with olanzapine experienced a significantly greater increase in body weight, culminating in consistently higher body weights than those of the control group by the study’s conclusion (Figures 1A, B). The administration of olanzapine was distinctly linked to an accelerated average body weight gain, markedly exceeding that observed in the control group.

To investigate the central abnormalities induced by olanzapine-induced obesity, we first used fUS to screen for resting-state functional connectivity in various brain regions. We found that awake fUS analyses offered deeper insights, where we delineated the LS into its caudal (LSc), rostral (LSr), and ventral (LSv) parts following the Allen Mouse Brain Common Coordinate Framework. We investigated the functional connectivity with key hypothalamic regions: the lateral hypothalamic area (LH), the anterior hypothalamic nucleus (AHN), and the paraventricular nucleus (PVH) (Figure 1C). In addition, for a broader perspective, we contrasted LS connectivity with other salient brain regions captured within the same fUS scan slice, such as the Anterior cingulate cortex (ACC), the hippocampal CA3 region (CA3), the Anterior group of the dorsal thalamus (ADth), the Ventral group of the dorsal thalamus (VDth), and the Posterior amygdalar nucleus (PA) (Figure 1D). Our analyses revealed group-dependent variations in these connectivities (Figures 1E, F), notably an increase in the functional connectivity z of LSc-PVH in the olanzapine group, while the z of LSv-PVH was significantly reduced. Our study further revealed that olanzapine’s influence extends beyond LS-hypothalamic functional connectivity; it also modulates the functional interplay within the LS brain regions themselves. Specifically, we observed a significant enhancement in the functional connectivity between LSc and LSv in the group treated with olanzapine (Figure 1G). These findings indicate that chronic olanzapine administration modulates LS-hypothalamic functional connectivity.

Given the significant impact of olanzapine on both body weight gain and functional connectivity, particularly involving the LS and hypothalamus, our findings pave the way for deeper investigations into the LS’s role in mediating these effects. The substantial correlation between functional connectivity changes and weight gain underscores the necessity of further detailed analyses of the LS to uncover the underlying mechanisms of olanzapine’s metabolic side effects.

### 3.2 Body composition and biochemical parameters of mice

**FIGURE 2:**
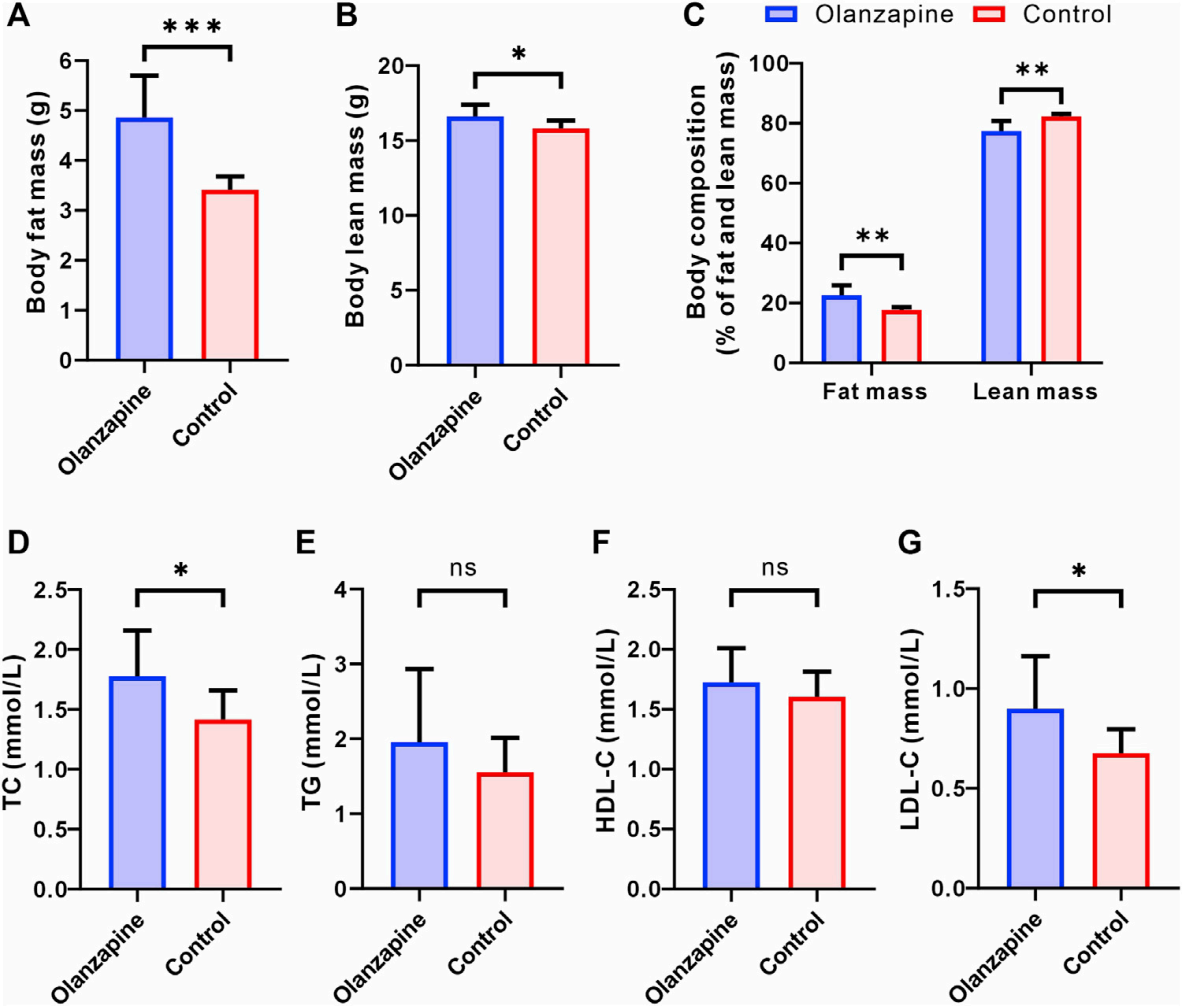
Results of statistical analysis for body composition and serum lipoprotein levels between two groups. N = 9 per group. **(A)** Body fat mass of mice after removal of the head. Welch’s t-test, F = 9.525, *p* = 0.0006, R^2^ = 0.7170, t = 4.948, df = 9.662. **(B)** Body lean mass of mice after removal of the head. Unpaired t-test. F = 2.128, *p* = 0.0214, R^2^ = 0.2890, t = 2.550, df = 16. **(C)** Body composition of mice after removal of the head. Welch’s t-test, F = 14.50, *p* = 0.0023, R^2^ = 0.6577, t = 4.181, df = 9.098. **(D)** Serum TC level measured at the end of 12 weeks treatment. Unpaired t-test. F = 2.503, *p* = 0.0295, R^2^ = 0.2632, t = 2.391, df = 16. **(E)** Serum TG level measured at the end of 12 weeks treatment. Welch’s t-test, F = 4.608, *p* = 0.2912, R^2^ = 0.0977, t = 1.107, df = 11.32. **(F)** Serum HDL-C level measured at the end of 12 weeks treatment. Unpaired t-test. F = 1.849, *p* = 0.3219, R^2^ = 0.0613, t = 1.022, df = 16. **(G)** Serum LDL-C level measured at the end of 12 weeks treatment. Welch’s t-test, F = 4.841, *p* = 0.0414, R^2^ = 0.3221, t = 2.304, df = 11.17. The data are shown as mean ± SD. *p < 0.05, **p < 0.01, ***p < 0.001.

Building on our findings regarding body weight and functional connectivity, we delved deeper into the metabolic implications of olanzapine treatment by assessing changes in body composition and lipid metabolism. Utilizing EchoMRI, we observed marked alterations in body composition among olanzapine-treated mice. Specifically, these mice exhibited a significant increase in both the absolute fat mass and its proportion of the total body mass. Interestingly, while absolute lean mass was higher in the olanzapine group, its proportion relative to total body mass was significantly diminished, highlighting the distinct impact of olanzapine on body composition (Figures 2A–C).

Serum lipid profile analysis further elucidated olanzapine’s metabolic effects. By the study’s conclusion, olanzapine administration had notably elevated TC and LDL-C levels, signaling a shift towards less favorable lipid metabolism (Figures 2D, G). Contrarily, levels of TG and HDL-C did not show significant differences between the olanzapine and control groups (Figures 2E, F).

These combined results not only corroborate the significant influence of olanzapine on body weight and functional brain connectivity but also reveal its profound effects on body composition and lipid profiles. The notable increase in fat accumulation and adverse shifts in lipid metabolism underscore the comprehensive metabolic alterations induced by olanzapine treatment. Such findings underscore the critical need for further detailed exploration of the LS area to uncover the mechanistic pathways through which olanzapine exerts these multifaceted metabolic effects.

### 3.3 RNA-seq analysis of the LS region

To elucidate the impact of olanzapine on metabolic side effects, including weight gain, within the LS area, we performed RNA-seq analysis on samples from two groups of mice. Our goal was to identify differentially expressed genes (DEGs) associated with the metabolic phenotypes induced by olanzapine treatment.

Employing a rigorous data processing and analysis workflow, we obtained between 50.22 and 54.81 million raw reads per sample. Quality filtering preserved approximately 47.96–50.04 million clean reads for each sample. Successful alignment with the reference genome was achieved, demonstrating a mapping rate of 98.16%–98.31%, which enabled the calculation of FPKM values for 35,415 genes. A stringent selection criterion—Q value <0.05 and a fold change >2 or <0.5—facilitated the identification of 735 significantly DEGs.

**FIGURE 3:**
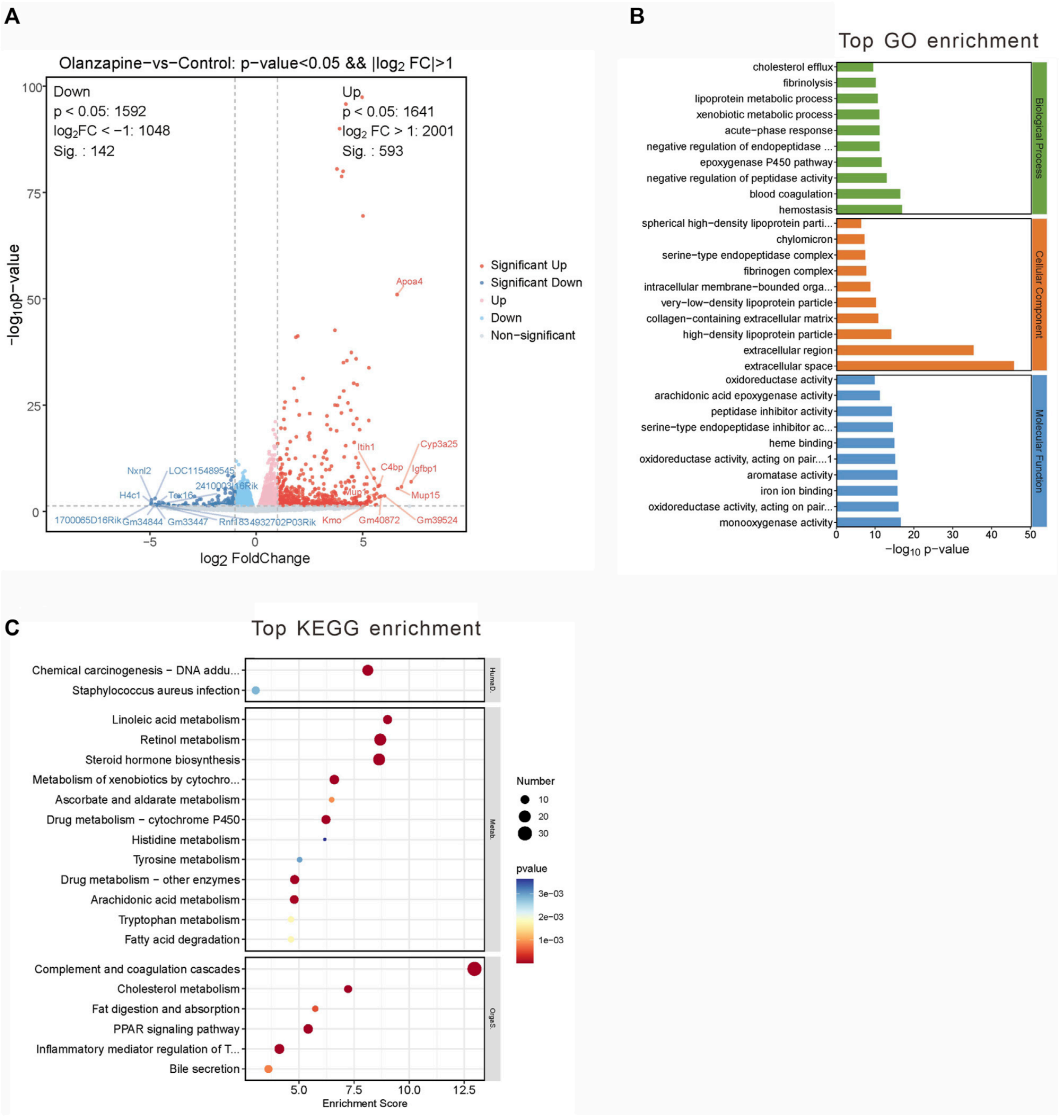
DEGs between two groups and gene analysis of DEGs. **(A)** Volcano plot of DEGs. Red represents 593 upregulated genes and blue represents 142 downregulated genes with |log_2_(FC)|>1 and *p* < 0.05. **(B)** Top 10 GO terms related to biological processes, cellular component and molecular function. **(C)** KEGG enrichment analysis of MEblue module. Terms are arranged by *p*-value. The number of gene types is represented by the gene number.

Among these, 593 genes were found to be upregulated, and 142 genes downregulated in the olanzapine group compared to controls (Figure 3A). Subsequent pathway enrichment analysis of these DEGs revealed notable findings. GO analysis indicated significant enrichment in biological processes such as lipoprotein metabolic processes and cholesterol efflux. Cellular component analysis highlighted enrichment in entities like high-density lipoprotein particle, very-low-density lipoprotein particle, and chylomicron. Furthermore, molecular function analysis pointed to an enrichment in functions including monooxygenase activity, aromatase activity, and arachidonic acid epoxygenase activity (Figure 3B). KEGG pathway analysis revealed enrichment in pathways critical to lipid metabolism, such as linoleic acid metabolism, PPAR signaling, and cholesterol metabolism (Figure 3C).

These findings underscore olanzapine’s influence on the expression of lipid metabolism-related genes within the LS brain regions, suggesting a molecular basis for the metabolic side effects observed. To fully understand the implications of these alterations, it is essential to integrate these data with the comparative analysis of the olanzapine and control groups.

### 3.4 WGCNA applied to RNA-Sequencing data

**FIGURE 4:**
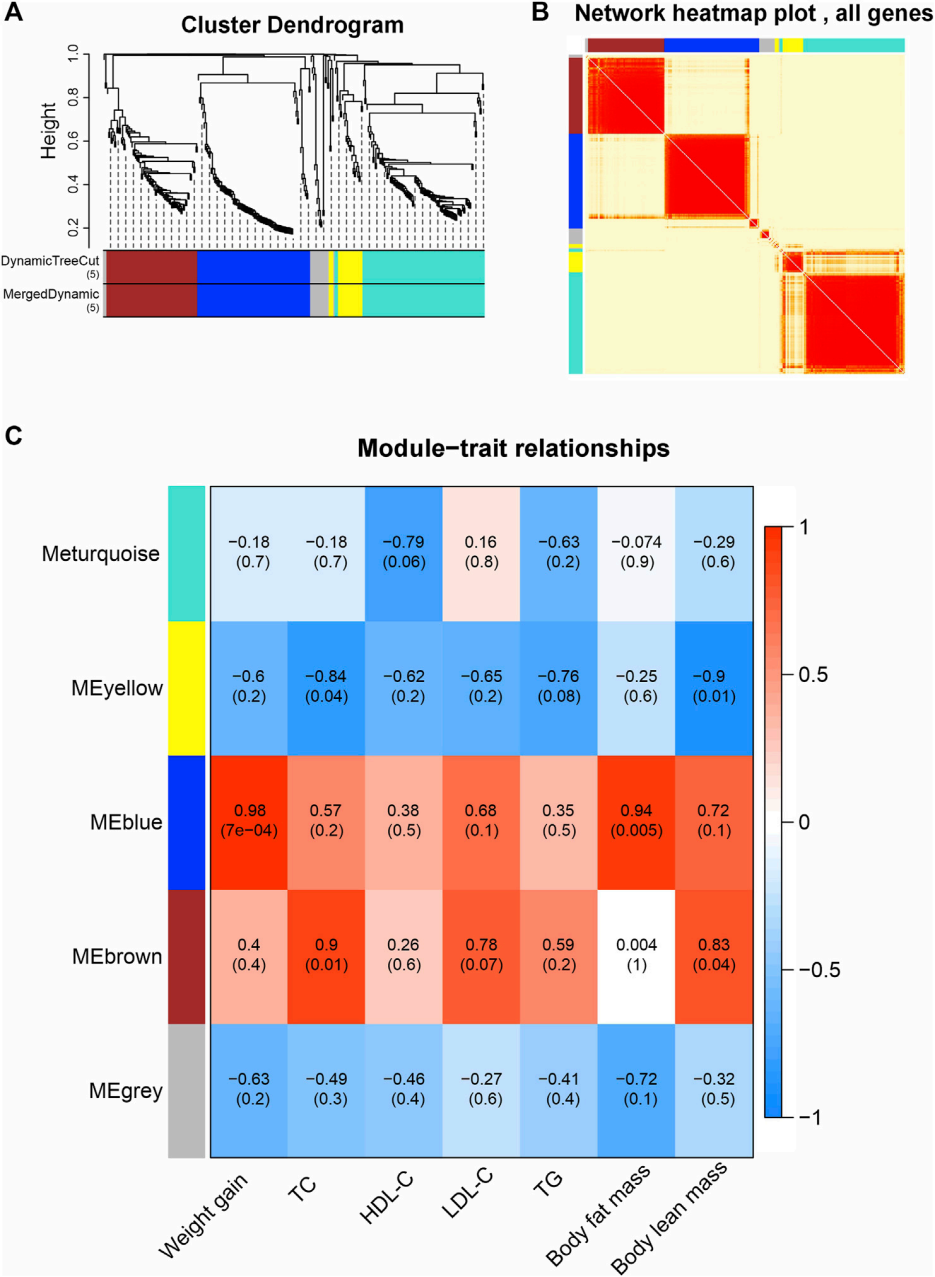
Weighted gene co-expression network analysis (WGCNA). **(A)** Average connectivity analysis for different soft threshold powers. **(B)** Heatmap of correlations among WGCNA modules. **(C)** Heatmap of correlations between sample traits and WGCNA module eigengene. Upper values in each table cell indicate correlations. *p* values for association testing are the lower values in brackets.

To bridge the gap between RNA-seq data and the metabolic alterations caused by olanzapine, we employed WGCNA. This sophisticated approach enabled us to construct co-expression networks from the FPKM values, effectively grouping genes into five distinct modules based on their expression patterns: MEturquoise, MEblue, MEbrown, MEyellow, and MEgrey (Figures 4A, B). The MEgrey module consisted of genes not significantly correlated with any specific metabolic trait, serving as a contrast to the other more functionally targeted modules.

We then explored the correlation of module eigengenes (MEs) with various phenotypic traits linked to olanzapine’s metabolic effects (Figure 4C). The MEblue module emerged as particularly noteworthy, showing a strong association with both weight gain and body fat mass (R = 0.98, *p* = 0.0007; R = 0.94, *p* = 0.005). This finding highlights the MEblue module’s central role in the observed weight and fat accumulation. On the other hand, the MEbrown module displayed a significant correlation with TC levels and body lean mass (R = 0.9, *p* = 0.01; R = 0.83, *p* = 0.04), revealing distinct gene networks that may drive specific facets of the metabolic changes induced by olanzapine.

Our WGCNA findings pinpoint critical gene networks within the MEblue and MEbrown modules as central to olanzapine’s metabolic side effects. This sets the stage for an in-depth exploration of these modules to understand the specific mechanisms at play.

### 3.5 Analysis of the MEblue module

The MEblue module, comprising 97 genes, featured 84 DEGs that were upregulated in the olanzapine-treated group compared to controls. To unravel the biological significance of these genes, we conducted GO and KEGG enrichment analyses.

**FIGURE 5:**
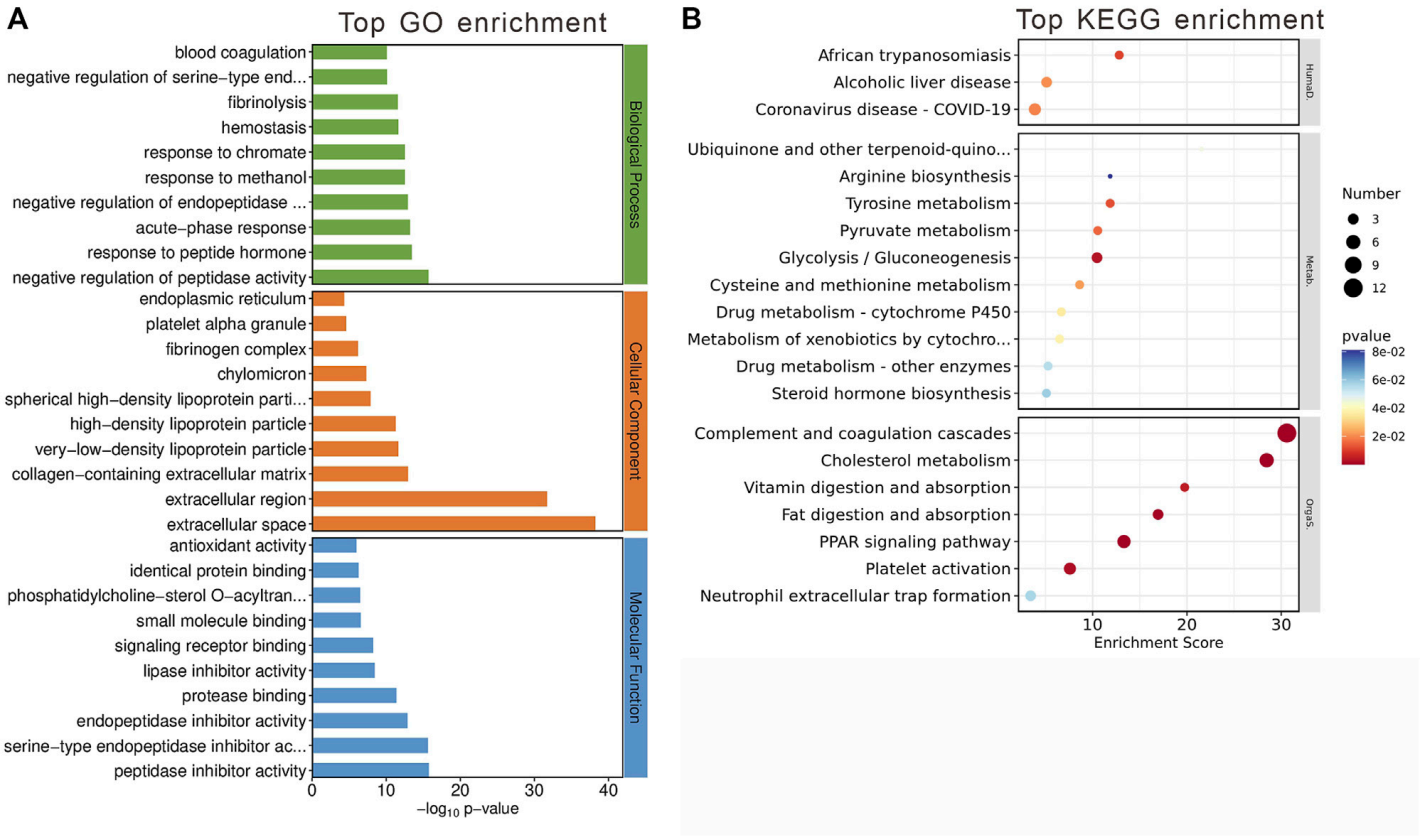
Gene Analysis for the MEblue module genes. **(A)** Top 10 GO terms related to biological processes, cellular component and molecular function. **(B)** KEGG enrichment analysis of MEblue module. Terms are arranged by *p*-value. The number of gene types is represented by the gene number.

The GO analysis segmented these 84 genes into three main categories: biological processes, cellular components, and molecular functions (Figure 5A). The top GO keywords were chosen based on their *p*-values. The module genes provided information about potential biological processes such as negative regulation of peptidase activity, peptide hormone response, and acute-phase response. Identified cellular components pathway included very-low-density and high-density lipoprotein particles, among others. Molecular functions of interest were lipase inhibitor activity and signaling receptor binding. KEGG enrichment analysis revealed involvement in systemic functions like complement and coagulation cascades, cholesterol metabolism, and the PPAR signaling pathway, indicating the module’s potential impact on metabolic processes (Figure 5B).

These findings suggest that the MEblue module has a significant impact on metabolic processes altered by olanzapine treatment, offering insights into the molecular mechanisms driving these changes.

### 3.6 Analysis of the MEbrown module

Contained within the MEbrown module are 78 genes, of which 9 have been pinpointed as DEGs between the olanzapine-treated and control groups. Also, all identified DEGs within this module were upregulated following olanzapine treatment. To decipher the biological implications of these alterations, we conducted GO and KEGG enrichment analyses on these genes.

**FIGURE 6:**
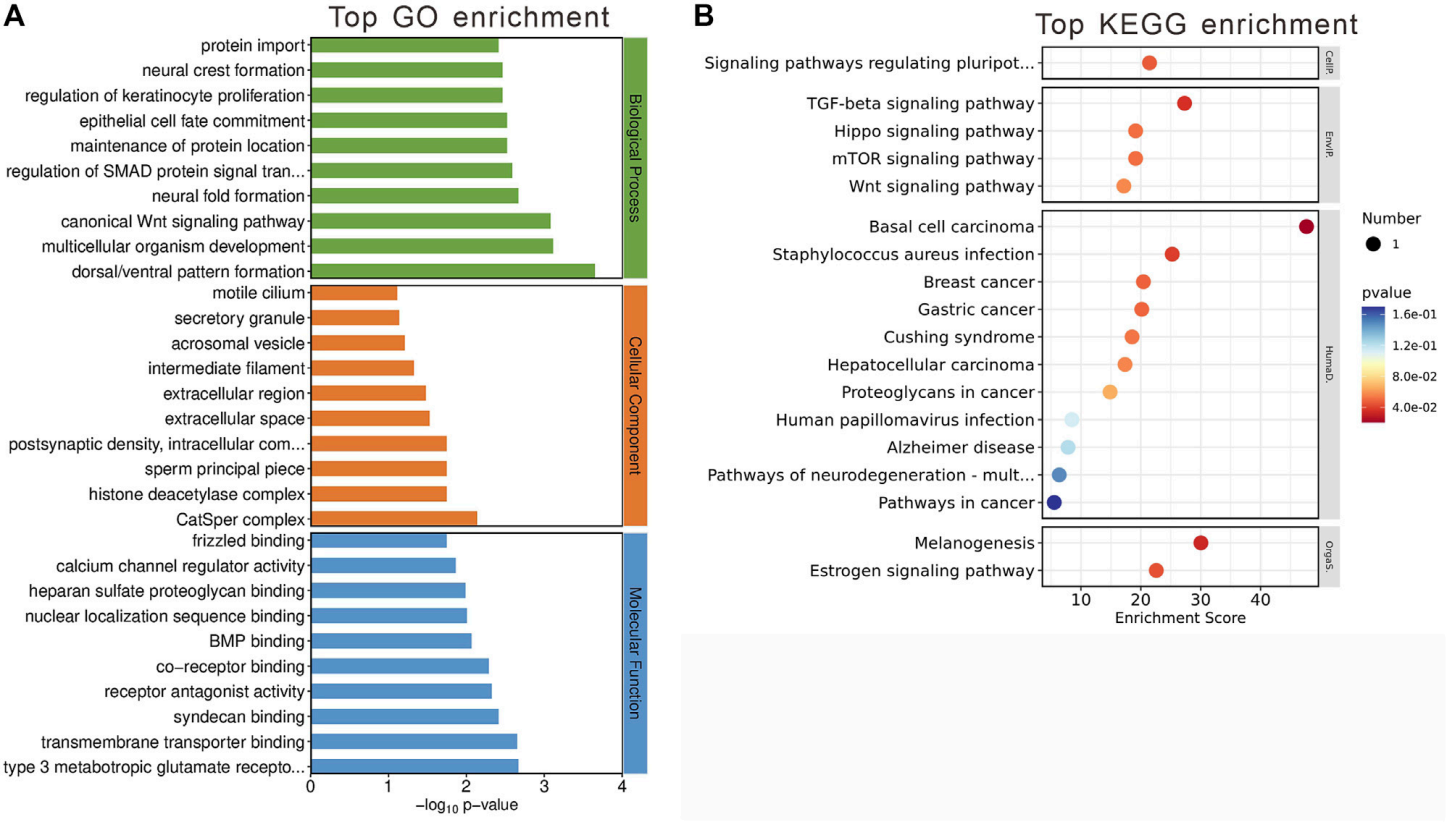
Gene Analysis for the MEbrown module genes. **(A)** Top 10 GO terms related to biological processes, cellular component and molecular function. **(B)** KEGG enrichment analysis of MEbrown module. Terms are arranged by *p*-value. The number of gene types is represented by the gene number.

The GO enrichment analysis unveiled these genes’ involvement in pivotal biological processes, notably including canonical Wnt signaling and the regulation of SMAD protein signal transduction. When examining cellular components, our analysis highlighted significant associations with the postsynaptic density and various intracellular components. In terms of molecular functions, a noteworthy finding was the genes’ interactions with type 3 metabotropic glutamate receptors, underscoring their potential role in neurotransmission and neuronal signaling (Figure 6A).

KEGG enrichment analysis further elucidated the module’s significance, revealing involvement in critical signaling pathways such as TGF-beta and estrogen signaling pathways (Figure 6B). These pathways are crucial for a wide range of cellular functions, suggesting that the genes within the MEbrown module play roles in complex signaling networks that could be linked to the metabolic and neurological effects observed with olanzapine treatment.

### 3.7 Analysis of target genes within MEblue and MEbrown modules

**FIGURE 7:**
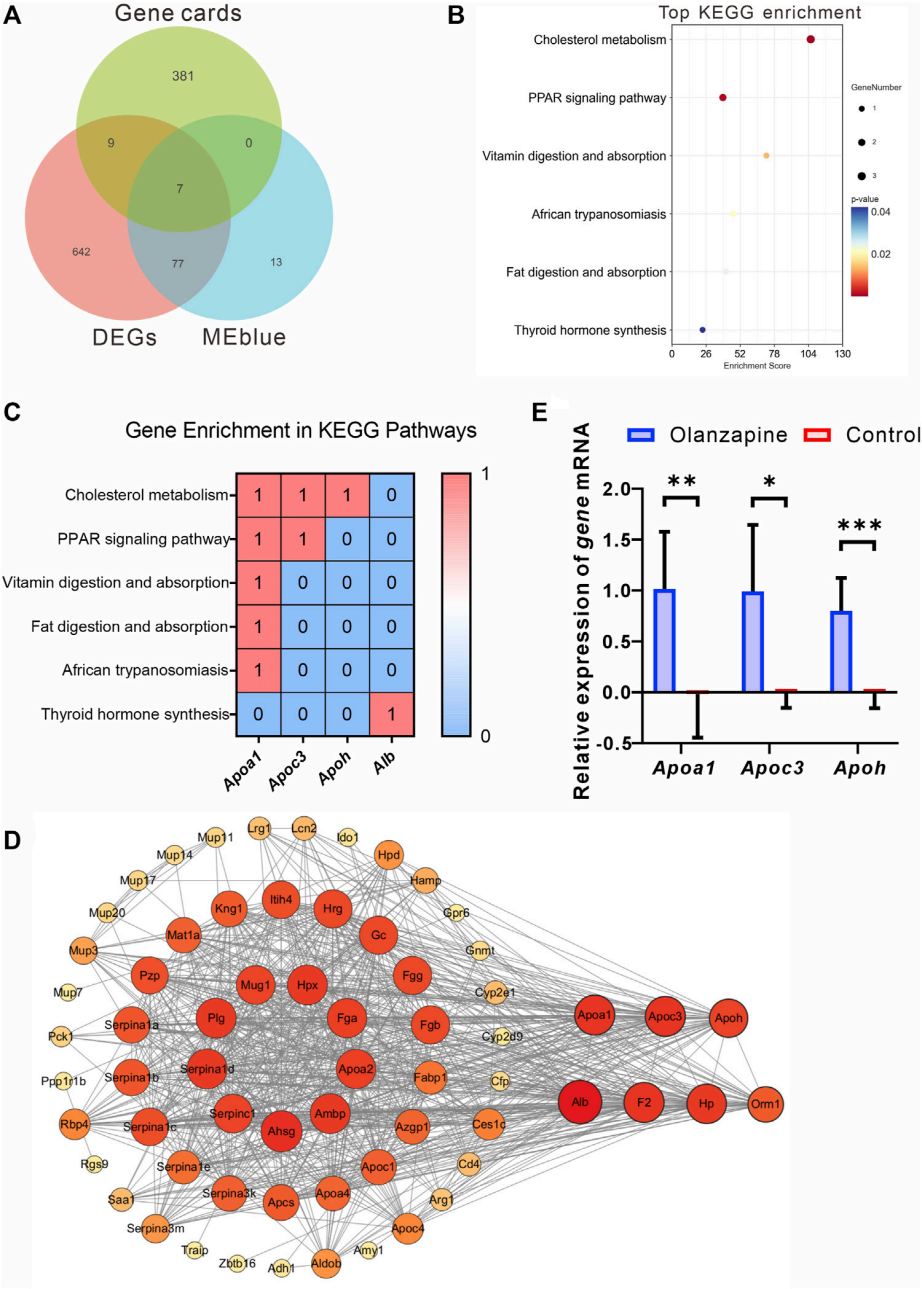
Identification, analysis and qRT-PCR validation of key genes. **(A)** Venn diagram showing the genes from Genecards, MEblue and DEGs. **(B)** The top terms of KEGG enrichment analysis sorted by the *p*-value for overlapping genes in Genecards, MEblue and DEGs. The number of gene types is represented by the gene number. **(C)** Heatmap of association between Apoa1, Apoc3, and Apoh with lipid metabolism pathways. Values in each table cell indicate whether gene was enriched in this KEGG pathway (1) or not (0). **(D)** The PPI network of the 65 overlapping genes. The node color and size represent the magnitude of the degree value. **(E)** The level of *Apoa1* (Mann-Whitney U test, *p* = 0.0022), *Apoc3* (Welch’s t-test, F = 18.90, *p* = 0.0128, R^2^ = 0.7034, t = 3.620, df = 5.528), and *Apoh* (Unpaired t-test. F = 4.474, *p* = 0.0003, R^2^ = 0.7535, t = 5.529, df = 10. mRNA expression in the lateral septum area. N = 6 per group. The data are shown as mean ± SD. *p < 0.05, **p < 0.01, ***p < 0.001.

To identify crucial genes within the MEblue and MEbrown modules potentially contributing to olanzapine’s metabolic side effects, we cross-referenced the modules’ DEGs with 397 olanzapine target genes listed in the GeneCards database. Among these, seven genes were identified as common between the olanzapine targets and the DEGs of the MEblue module, all showing upregulation in response to olanzapine treatment (Figure 7A). These overlapping genes were significantly enriched in key metabolic pathways, including cholesterol metabolism, PPAR signaling, and fat digestion and absorption. Notably, *Apoa1*, *Apoc3*, and *Apoh* emerged as genes of particular interest due to their roles in lipid metabolism (Figures 7B, C).

Leveraging the STRING online tool, we developed a protein-protein interaction (PPI) network for the 84 genes bridging the MEblue module and DEGs. Analyzed using Cytoscape 3.10.1, this network initially featured 65 nodes and 788 edges, illustrating the extensive interconnectivity among these genes. Within this network, *Apoa1*, *Apoc3*, and *Apoh* stood out for their high degree of connectivity, suggesting a central role in the module’s biological significance (Figure 7D).

To corroborate the RNA-seq findings, we conducted qRT-PCR analyses to validate the transcriptional profiles of *Apoa1*, *Apoc3*, and *Apoh* in LS tissues from olanzapine-treated mice. The mRNA expression levels of these genes were significantly higher in the olanzapine group compared to controls, consistent with the RNA-seq data. This validation highlights olanzapine’s influence on the expression of genes critical to lipid metabolism and supports their involvement in the drug’s metabolic effects.

In contrast, the overlap between olanzapine targets and the MEbrown module yielded five genes. However, none exhibited differential expression between the olanzapine and control groups. This suggests unique regulatory mechanisms or roles in response to olanzapine that may not involve changes in gene expression.

In summary, our analysis has elucidated a subset of olanzapine’s target genes within the MEblue module that are significantly associated with metabolic pathways, particularly lipid metabolism. The validation of these genes’ expression profiles, notably *Apoa1*, *Apoc3*, and *Apoh*, underscores their potential role in olanzapine-induced metabolic alterations. While the analysis of the MEbrown module revealed a different set of target genes, the lack of differential expression suggests that alternative mechanisms are involved.

## 4 Discussion

In line with previous findings, long-term olanzapine treatment led to hyperphagia, obesity, and insulin resistance (Luo et al., 2021; Burschinski et al., 2023). Our st udy aimed to investigate whether olanzapine administration not only improves central psychiatric symptoms but also affects central regulatory neural projections linked to obesity onset. Consequently, we examined key neural circuits impacted by chronic olanzapine treatment, revealing modulation of functional connectivity between the LH and LS brain regions.

Additionally, we observed altered gene expression in the LS region of mice. Through RNA-seq and WGCNA, we identified a gene module closely associated with increased body weight and adiposity, prominently featuring genes involved in lipid metabolism pathways, such as *Apoa1*, *Apoc3*, and *Apoh*. These findings suggest that olanzapine influences body weight and adiposity by affecting lipid metabolism-related genes in the LS brain region and altering its functional connectivity with the LH.

In CNS, olanzapine interfere profoundly with glucose and lipid homeostasis acting mostly on hypothalamus (Carli et al., 2021). Distubances of neuronal activity and axon projection were induced by olanzapine treatment (Kim et al., 2022). Furthermore, modulation of hypothalamic AMPK signaling by olanzapine controls energy balance and body weight. Given that homeostatic inputs from the hypothalamus are integrated with hedonic feeding signals from mesolimbic pathways and signals from superordinate regions to generate an orchestrated response in feeding behavior, glucose metabolism, and energy homeostasis regulation, We conducted fUS to identify the LS as a key brain region with functional connectivity to fUS.Two key neural pathways between the LS and the hypothalamus, from the paraventricular nucleus of the hypothalamus to the ventral LS, and from the dorsal LS to the lateral hypothalamic area, are implicated in both physiological and psychological functions. (Carus-Cadavieco et al., 2017; Xu et al., 2019; Mu et al., 2020). Furthermore, recent studies highlight that extended inhibition of projections from melanocortin-4 receptor-expressing neurons in the paraventricular hypothalamus to the LS may precipitate obesity due to reduced energy expenditure (Xu et al., 2023b). We observed a significant alteration in the functional connectivity between the LS and the hypothalamus in mice treated with olanzapine, particularly in the neural projections between the PVH and the LS, indicating substantial neurophysiological changes. Although not every pathway from the LS to the hypothalamus showed significant alteration, the overall trend in connectivity suggests a broad, possibly cumulative, impact of long-term olanzapine administration on these critical neural circuits. At the same time, the altered functional connectivity between the PVH and different regions of the LS was not consistent, also reflecting the more complex effects of olanzapine on this neural circuit.

The initiation of our discussion delves into the pivotal role of the LS area within the central nervous system, focusing on its established significance in regulating metabolic and behavioral processes. The LS emerges as a central hub orchestrating a range of emotions such as reward, feeding, anxiety, fear, sociability, and memory (Rizzi-Wise and Wang, 2021). Evidence indicates the LS’s integral role in various facets of feeding behavior, including the motivation to seek food, actual food consumption, and taste preference. The motivation for food, reflecting the pursuit rather than the act of eating, has been studied in relation to the function of specific receptors in the LS. Beyond its impact on food motivation, the LS also directly influences food consumption through the pharmacological modulation of various receptors. Therefore, in the current study, we explored the underlying mechanisms using a broad approach and performed global transcriptomic analysis of the LS, a valuable tool allowing for the accurate and unbiased identification of genes differentially regulated between treatments. Using this approach, we have identified LS genes altered in response to olanzapine treatment and have suggested both known and novel candidate genes that merit further investigation.

Our comprehensive exploration of olanzapine’s metabolic side effects has unveiled its substantial impact on body weight, functional brain connectivity, body composition, and lipid metabolism, systematically revealing the depth of olanzapine’s influence on metabolic processes. In our study, C57BL/6 mice subjected to a diet containing olanzapine demonstrated significant body weight gain, particularly noted in an increased fat mass ratio. This finding highlights the drug’s potent effect on promoting weight gain, an adverse outcome frequently observed in clinical settings. The pronounced increase in body weight and fat mass underscores the urgent need to understand the mechanisms driving these changes, aiming to mitigate such undesirable effects in patients undergoing olanzapine treatment. The administration of olanzapine also resulted in notable changes in body composition, characterized by an increase in fat mass and alterations in serum lipid profiles, with elevated TC and LDL-C levels. These alterations in lipid metabolism provide a clearer picture of how olanzapine disrupts metabolic homeostasis, further supported by our detailed RNA-seq analysis.

In this study, we found 735 genes significantly altered in the LS following olanzapine treatment, including previously identified genes such as *Apoa1*, *Soat2*, and *Crp*, which are closely related to lipid transport, cholesterol synthesis, and inflammation (Pramfalk et al., 2022; Endres et al., 2024). We observed changes in a variety of genes regulating energy, with a notable increase in apolipoprotein-related genes such as *Apoa1*, *Apoa2*, *Apob*, *Apoc3*, *Apoh* and *Apom*. Through RNA-seq analysis of the LS region, we identified a robust set of DEGs, indicating a shift towards less favorable lipid metabolic pathways. The application of WGCNA allowed us to dissect these effects at a molecular level, pinpointing the MEblue and MEbrown modules as central to the observed metabolic changes. Specifically, the MEblue module’s association with weight gain and fat accumulation, enriched in cholesterol metabolism and PPAR signaling pathways, underscores olanzapine’s impact on lipid homeostasis (Pinna, 2023). Conversely, the MEbrown module’s link to canonical Wnt signaling and SMAD protein signal transduction regulation points towards olanzapine’s broader effects on cellular signaling networks (Chambers et al., 2009; Kim et al., 2021).

Crucially, our analysis of target genes within these modules, particularly the enrichment of *Apoa1*, *Apoc3* and *Apoh* in key metabolic pathways, validates the RNA-seq findings and underscores their potential role in mediating olanzapine’s adverse metabolic outcomes. The absence of differential expression in the MEbrown module’s olanzapine targets suggests distinct regulatory mechanisms or roles in response to olanzapine, inviting further investigation. The significant roles of apolipoprotein A-Ⅰ (APOA-Ⅰ), apolipoprotein-Ⅲ (APOC-Ⅲ) and apolipoprotein H (APOH) in lipid metabolism are well-established, with these apolipoproteins participating in essential processes like cholesterol transport, triglyceride regulation, and innate immunity (Agar et al., 2011; Norata et al., 2015; Zewinger et al., 2020; Giammanco et al., 2023).

Recent advancements have highlighted the significance of apolipoproteins in the central nervous system (CNS), particularly due to the brain’s high lipid content and reliance on internal cholesterol synthesis, necessitated by the blood-brain barrier which limits external lipid entry (Björkhem and Meaney, 2004). This has led to the discovery of distinct apolipoprotein compositions and regulatory mechanisms within the CNS, separate from peripheral systems. The critical role of central apolipoproteins in lipid metabolism and immune regulation within the CNS is becoming evident, given neurons’ sensitivity to immune disruptions and their dependency on consistent lipid supplies (Endres, 2021). This role is further underscored by their contribution to neurological disease pathogenesis. Central apolipoproteins, some originating peripherally and others synthesized locally (e.g., APOA-I in brain endothelial cells, APOH in astrocytes and neurons), are implicated in neurodegenerative disorders such as Parkinson’s disease and Alzheimer’s disease (AD) (Elliott et al., 2010; Xu et al., 2023a). At the same time, some researches focus on the role of central lipid metabolism in modulating systemic metabolism and obesity. Hypothalamic astrocytes, influenced by angiopoietin-like four and peroxisome proliferator–activated receptor gamma, regulate fatty acid homeostasis, which impacts neuronal responses to metabolic changes and protects against diet-induced obesity (Varela et al., 2021). This brain region also signals nutritional status to peripheral tissues like the liver, affecting glucose production, lipogenesis, and TG secretion through fatty acid sensing and autonomic nervous system activity (Bruce et al., 2017). These mechanisms underscore the potential of targeting central lipid metabolism to treat metabolic disorders.

Research in schizophrenia has shown a notable decrease in APOA-I across five tissues (cerebrospinal fluid, brain, liver, red blood cells, and serum) in patients, potentially contributing to their heightened risk of metabolic disorders (Huang et al., 2008). Consequently, some researchers propose using metabolic syndrome counteracting drugs to mitigate antipsychotics’ weight and metabolic side effects by raising APOA-I levels (Xiang et al., 2019). Contrarily, other studies indicate antipsychotic usage increases APOA-I levels in serum and cerebrospinal fluid, correlating with rises in body mass index, TC, and TG (Martins-De-Souza et al., 2010; Song et al., 2014). Variations in study samples and antipsychotic medications used may explain these divergent findings.

Simultaneously, research has highlighted that lipoprotein metabolism’s role extends beyond the metabolic side effects in schizophrenia to include the disorder’s psychiatric symptoms. For instance, a study identified a strong link between low HDL serum levels and increased aggression in female schizophrenia patients (Herceg et al., 2022). In contrast, another study observed that rising serum HDL levels during the first year of antipsychotic treatment corresponded with reduced negative symptoms (Gjerde et al., 2018). These insights indicate that lipid metabolism, particularly within the central nervous system, may influence schizophrenia’s psychiatric symptoms and could be a target for antipsychotic medications. Furthermore, it is speculated that antipsychotic-induced alterations in central lipid metabolism might contribute to their metabolic side effects, such as weight gain. This intertwining of lipid metabolism with psychiatric manifestations, especially in schizophrenia, opens an intriguing perspective on the comprehensive effects of antipsychotic treatments. The alterations in apolipoprotein gene expression induced by olanzapine may serve as a double-edged sword, affecting both the metabolic profile and psychiatric symptoms of schizophrenia patients. This dual influence calls for an integrated treatment approach that addresses the multifaceted nature of schizophrenia, balancing psychiatric symptom management with metabolic health.

The upregulation of apolipoprotein genes, including *Apoa1*, *Apoc3*, and *Apoh*, in the olanzapine-treated group unveils a profound modulation of lipid metabolism, reminiscent of their known peripheral roles yet extending this regulatory capacity deep into the neural circuits of the brain. This observation not only underscores the direct effect of olanzapine on CNS lipid metabolism but also hints at a potential mechanism contributing to the drug’s metabolic side effects.

## 5 Conclusion

In this study, the reduced functional connectivity between the LS and the hypothalamus observed via fUS imaging enriches our understanding of olanzapine’s neural impact. These findings, combined with the molecular insights into lipid metabolism gene expression, suggest a comprehensive model wherein olanzapine’s effects permeate from molecular to systemic levels, affecting both brain functionality and overall metabolic regulation. The insights garnered from this research pave the way for future investigations aimed at dissecting the specific mechanisms by which olanzapine modulates lipid metabolism in the brain. Changes in central lipid metabolism may contribute to the weight gain and metabolic side effects associated with olanzapine treatment. This study offers valuable insights for future research into olanzapine’s precise mechanisms in modulating brain lipid metabolism.

## Data Availability

The data presented in the study are deposited in the Gene Expression Omnibus (GEO) repository, accession number GSE268867.

## Footnotes

1 GeneCards database (https://www.genecards.org, version 5.0)

2 STRING database (https://string-db.org, version 11.5)
